# Hybrid generative adversarial network based on frequency and spatial domain for histopathological image synthesis

**DOI:** 10.1186/s12859-025-06057-9

**Published:** 2025-01-27

**Authors:** Qifeng Liu, Tao Zhou, Chi Cheng, Jin Ma, Marzia Hoque Tania

**Affiliations:** 1 Centre for Big Data Research in Health, University of New South Wales, Sydney, Australia; 2 Department of Respiratory and Critical Medicine, The Second Affiliated Hospital of Nanchang University, Nanchang, China; 3 School of Computer Science and Engineering, University of New South Wales, Sydney, Australia; 4 Faculty of Engineering, The University of Sydney, Sydney, Australia

**Keywords:** Generative adversarial networks, Cross-attention mechanism, Spatial domain, Frequency domain, Histological slide images, Variable-window mixed attention, Spectral filtering, Image generation fusion, Transformer

## Abstract

**Background:**

Due to the complexity and cost of preparing histopathological slides, deep learning-based methods have been developed to generate high-quality histological images. However, existing approaches primarily focus on spatial domain information, neglecting the periodic information in the frequency domain and the complementary relationship between the two domains. In this paper, we proposed a generative adversarial network that employs a cross-attention mechanism to extract and fuse features across spatial and frequency domains. The method optimizes frequency domain features using spatial domain guidance and refines spatial features with frequency domain information, preserving key details while eliminating redundancy to generate high-quality histological images.

**Results:**

Our model incorporates a variable-window mixed attention module to dynamically adjust attention window sizes, capturing both local details and global context. A spectral filtering module enhances the extraction of repetitive textures and periodic structures, while a cross-attention fusion module dynamically weights features from both domains, focusing on the most critical information to produce realistic and detailed images.

**Conclusions:**

The proposed method achieves efficient spatial-frequency domain fusion, significantly improving image generation quality. Experiments on the Patch Camelyon dataset show superior performance over eight state-of-the-art models across five metrics. This approach advances automated histopathological image generation with potential for clinical applications.

## Introduction

Histopathology is a specialized field in medicine that primarily studies microscopic structural changes in tissues under pathological conditions. By observing diseased tissues, it helps experts diagnose diseases, determine the nature of the lesions, and formulate treatment plans. Histopathologists usually use microscopes to examine tissue samples taken from patients. Through a series of complex preprocessing steps [1] such as fixation, dehydration, paraffin embedding, staining, and sectioning, thin tissue slides are ultimately prepared for microscopic observation. However, obtaining tissue slides is a complex process involving many factors. Firstly, the preparation of tissue slide samples is very intricate. Each step, including fixation, dehydration, paraffin embedding, and staining, requires precise control. Any operational errors can lead to tissue degradation, shrinkage, deformation, damage, and reduced cell structure recognizability due to improper staining [2]. Secondly, the availability of tissue samples for slide preparation is very limited, especially for precise biomarker studies and rare disease research [3], which restricts histopathologists’ ability to study rare diseases. Lastly, obtaining high-resolution histological images requires not only high-quality tissue slides but also advanced imaging equipment [4], which entails high costs.

In recent years, some deep learning-based generative methods have effectively addressed these challenges. These models can use complex data-driven methods to generate high-quality tissue slides images, particularly outstanding in expanding tissue slides datasets corresponding to rare diseases. These models not only increase the number of images available for research and education but also enhance pathologists' understanding of the diagnosis and treatment strategies for these diseases.

Generative adversarial network (GAN) methods [5] have been widely used for histological slide image generation. Dolezal et al. [6] proposed a conditional GAN-based generative model to synthesize realistic, category-specific histological images to improve the interpretability of classifiers, enhancing the accuracy of pathologists in classifying rare tumor subtypes. Levine et al. [7] utilized GANs to generate high-resolution pathological images of 10 cancer tissue types, supplementing small training sets for cancer image classification, and validated the effectiveness of synthetic datasets for cancer classification diagnosis. Although these methods have enriched the diversity of tissue slide data, they still face limitations in the reliability and variety of generated images, such as mode collapse and the occurrence of blurriness and artifacts in the generated images.

Methods based on Variational Auto-Encoders (VAEs) [8] and diffusion models [9] have achieved remarkable progress. For example, Guleria et al. [10] proposed a histological image reconstruction method using variational auto-encoders and denoising variational auto-encoders, which significantly improved the classification accuracy of convolutional neural network-based classifiers. Moghadam et al. [11] introduced a diffusion probabilistic model incorporating prioritized morphology weighting and color normalization to synthesize high-quality neuropathological images of brain cancer. Shrivastava et al. [12] employed a conditional diffusion model to develop a method for synthesizing high-quality, realistic tissue samples with precise nuclear localization based on semantic instance masks of different nuclear types.

In this paper, unlike previous deep learning methods that focus solely on the spatial domain, we propose an unconditional generative adversarial network based on the fusion of spatial and frequency domain features. We designed a novel window attention mechanism [13] to capture the characteristics of images in the spatial domain and adopted a feature extraction module based on Fast Fourier transformation to capture the periodic information and intensity features in the frequency domain. However, simply concatenating spatial and frequency domain features fails to effectively capture critical information in the image. Therefore, we propose a novel cross-attention feature fusion module, which initially fuses features from both domains and then applies cross-attention mechanisms between the fused features, spatial domain features, and frequency domain features. This allows each domain to not only retain its own important information but also be guided by the attention from the other domain to optimize feature extraction. Through this cross-attention mechanism, the model can focus on the most relevant features, eliminate redundant information, and enhance the expressive power of the features. This approach significantly improves the clarity and detail of the generated images.

**Fig. 1.**
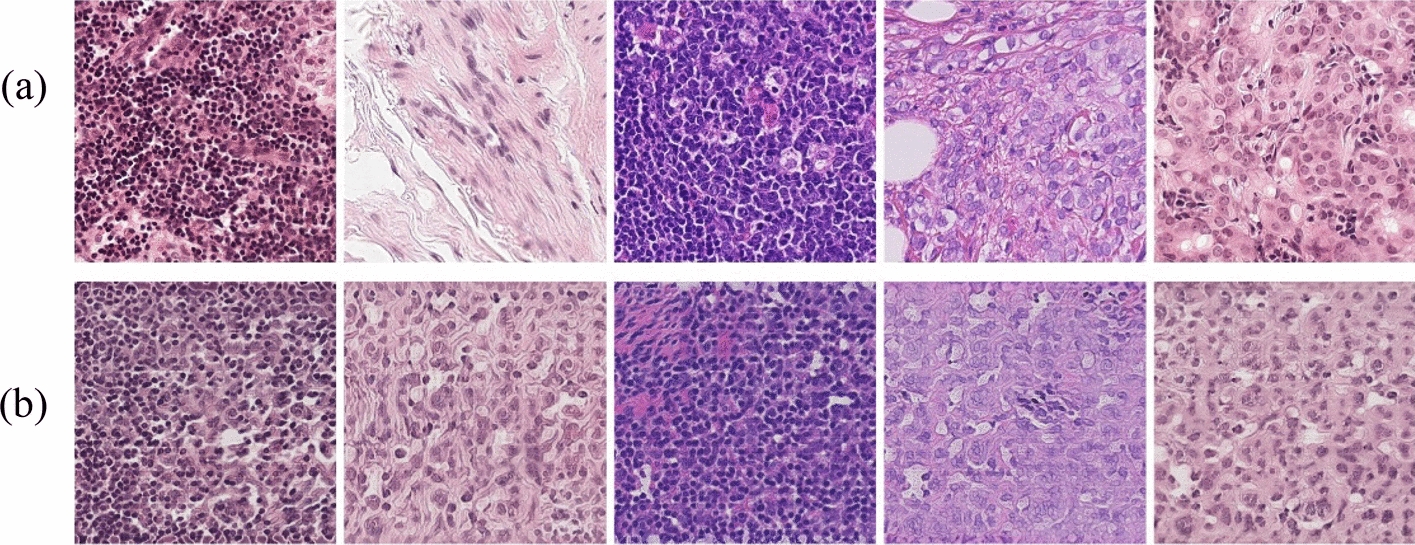
Comparison of real and generated images, **a** represents real histological slide images, and **b** represents images generated by our method. The generated tissue slide images are very similar to the real images in terms of color, detail, and cellular structure

Surprisingly, the proposed method achieved promising results, Fig. 1 provides an example. Our approach outperforms in both detail and overall structure, overcoming the limitations of conventional techniques in generating high-quality images. The main contributions of this paper are as follows:

- We propose a feature fusion method that combines spatial and frequency domain information. By simultaneously leveraging the advantages of both domains, it complements their roles in the feature extraction modules and is guided by a cross-attention mechanism, enabling the generation of high-quality pathological tissue slide images.
- We propose a hybrid feature extraction module based on the local window attention mechanism and the multi-scale window attention mechanism. The local window attention mechanism captures subtle lesions or cellular structure details in tissue slide images, while the multi-scale window attention mechanism dynamically adjusts the focus area size according to the processed image content. This allows the network to expand its focus to capture the global context when necessary or narrow its view to capture fine features. By combining these two mechanisms, our model achieves a comprehensive observation from microscopic details to macroscopic structures, significantly enhancing the overall quality of the generated images.
- We propose a frequency domain feature extraction module based on Fourier Transform [14]. This module converts data from the spatial domain to the frequency domain, effectively capturing and analyzing the periodic and structural features in the images. This process enhances the network's understanding of fine textures and global information in the images, significantly improving the quality and realism of the generated images.
- We utilize five evaluation metrics for generated image quality and no-reference image quality assessment metrics to conduct a multi-angle, comprehensive quality evaluation of the generated histological slide images, ensuring the comprehensiveness and reliability of the evaluation results.

The structure of this paper is as follows, the first section is the introduction, which elaborates on the current research status, research problems, and the bottlenecks and difficulties encountered in current research. The second section provides a detailed explanation of the latest methods for generating histological slide images. The third section explains in detail the various modules of the proposed method, including the overall architecture of the generator, the variable window mixing attention module, the spectral filtering module, the cross-attention fusion module, and the loss function. The fourth section describes the experiment settings and compares the proposed method against eight methods in three image generation quality metrics and two no-reference image quality assessment metrics. Ablation studies were conducted to verify the effectiveness of each module. The fifth section summarizes the paper based on the results, discusses the advantages and limitations of the proposed method, and outlines future research directions. Overall, this paper introduces an efficient approach for generating tissue slide images, further enhancing the quality, effectiveness, and diversity of the generated images, making them more suitable for the practical needs of medical research and diagnostic applications.

## Related work

Tissue slide images typically require high resolution and detail fidelity to accurately reflect minute tissue structures and pathological features. Additionally, the variety of staining agents used in tissue slide images leads to significant differences in staining effects. Furthermore, the diversity in tissue cell types and structures adds to the challenge of generating high-quality images. Currently, there are three categories of deep learning-based methods for generating tissue slide images: GAN-based methods, diffusion-based methods, and VAE-based methods. Additionally, fusion strategies can be effectively used in image generation by integrating features from different domains, leveraging their strengths to produce higher-resolution images with better detail and more accurate structures. The GAN-based methods generate high-quality tissue slide images through adversarial training between a generator and a discriminator. The diffusion model-based methods iteratively denoise and reconstruct noisy images, gradually restoring high-quality, high-detail fidelity tissue slide images over multiple iterative steps. The VAE-based methods encode input tissue slide images into a latent space probability distribution, decode this distribution back into the original image, and then sample new latent variables from the latent space, which the decoder then decodes into new tissue slide images. Fusion strategies involve integrating features from different methods or domains, leveraging their complementary strengths to generate images with enhanced resolution, detail fidelity, and more accurate representation of some structures. In the following sections, we provide a comprehensive overview of these three generative methods and fusion strategies, summarize the recent literature, and analyze each method.

### Methods based on generative adversarial networks

Methods based on GANs include generator and discriminator. The generator creates histological slide images from random noise, while the discriminator distinguishes between real histological slide images and generated ones. Through dynamic adversarial training, the generator continuously improves itself to produce high-quality images, and the discriminator enhances its ability to distinguish between real and generated images. These methods can generate histological slide images with rich details and natural textures, exhibiting strong adaptability and diversity. Recently, Xue et al. [15] proposed a conditional GAN utilizing class labels and designed a synthetic enhancement framework. By comparing the confidence levels of assigned labels to the feature similarity of actual labeled images, they selected synthetic image patches to generate synthetic images with high fidelity and diversity. To address the issue of class imbalance in the breast cancer histopathology image dataset, Saini et al. [16] proposed a novel network architecture using DCGAN [17] and VGG16 [18], using a transfer learning training strategy to enhance tissue slide datasets at different magnifications. Xue et al. [19] proposed a filtering mechanism to control the quality of selected synthetic image features and used a conditional GAN for data augmentation, improving the accuracy of classification models for cervical histopathology images. Since supervised deep learning methods are sensitive to domain shifts when dealing with tissue slides stained in different ways, their application is somewhat limited. Moreover, obtaining samples of images stained differently is both expensive and time-consuming. To address this, Vasiljević et al. [20] proposed a method for enhancing unsupervised image-to-image translation to generate datasets of the same tissue slides stained with different agents for image enhancement. Their analysis demonstrated the stain invariance of the generated images. Jiang et al. [21] introduced a multi-scale gradient GAN to synthesize rectal cancer tissue slide images. They utilized the pre-trained model to select fake images with high class probabilities to add to the training set, improving the performance of the classification model. However, GAN-based methods for generating medical tissue slides face a series of issues, including unstable training, mode collapse, and insufficient image details, limiting their widespread adoption and effectiveness in practical applications.

### Methods based on diffusion models

Methods utilizing diffusion models for generating tissue slide images involve a step-by-step denoising and reconstruction process, gradually generating high-quality images from noise. These methods can produce tissue slide images with high resolution and detail fidelity, and they demonstrate strong robustness to variations in staining methods and tissue types. Additionally, these methods can capture complex structures and diversity within the images, enhancing the reality and natural appearance of the generated images. Recently, Harb et al. [22] proposed a diffusion model approach for constructing histological slides from low resolution to high resolution, achieving gigapixel-scale whole slide image (WSI) generation. The results demonstrated that the generated WSI images closely match the structural features of actual WSI images. To address the impact of data imbalance on the performance of histopathology image classification, Guan et al. [23] introduced a post-discriminator mechanism-based diffusion model method for generating histopathology images. This method ensures the quality of augmented images by filtering synthetic images, thereby preventing poor-quality synthetic images from degrading subsequent classification performance and providing quality assurance for data augmentation. Zeng et al. [24] introduced a label diffusion graph learning method to enhance the recognition of breast cancer histology images using semi-supervised learning on small, labeled datasets. Yang et al. [25] proposed a dual-semantic diffusion model for generating high-quality and semantically related dynamic cellular imaging (DCI) images. This method combines semantic masks and reference images to generate DCI images that closely resemble real images, achieving higher accuracy in downstream segmentation tasks. Aversa et al. [26] proposed a hierarchical diffusion model that synthesizes segmentation masks to serve as conditions for generating tissue slide images, allowing the generation of tissue slides at any desired size and evaluating the plausibility of the generated data in downstream segmentation and classification tasks. However, the computational complexity of using diffusion models to generate tissue slide images is very high. The training process is time-consuming and requires significant computational resources. Additionally, tuning and training diffusion models is complex, often necessitating numerous experiments and expert knowledge to achieve optimal generation results.

### Methods based on variational auto-encoders

Methods based on VAEs generate tissue slide images by encoding the input tissue slide images into a latent space probability distribution and then decoding from that distribution back to the original images, thereby generating new tissue slide images. This approach can produce high-quality and diverse tissue slide images with high stability during training. Recently, Shwetha et al. [27] proposed a Gram-stained culture image generation network based on a vector quantized VAE combined with quality loss, achieving enhancement of the dataset. Lutnick et al. [28] introduced a VAE image generation method for augmenting mouse glomeruli tissue slide image datasets and used nonlinear dimensionality reduction to map these data to human glomeruli slide datasets, enhancing the human glomeruli slide training set. Tellez et al. [29] proposed a neural image compression method that converts gigapixel images into highly compact representations, improving convolutional neural network performance in label prediction for such images by transforming low-level pixel space information into higher-level latent space representations, thus enhancing model efficiency. To achieve color normalization for hematoxylin and eosin-stained tissue slide images, Zanjani et al. [30] proposed a VAE-based staining normalization method. This method performs color normalization on tissue slide images without requiring any data labels or assumptions, and the generated images outperform state-of-the-art methods in color constancy measurements. However, compared to other generative models, the quality of VAE-generated slide images is often lower, with less clear detail representation, which may result in blurriness and distortion. The continuous nature of the latent space can result in overly smooth generated images, lacking necessary variations and details, which is a significant limitation for medical images that require high precision.

### Fusion strategies for multi-domain feature integration

In recent years, fusion strategies have made significant progress in the field of image generation. By integrating information from different sources or feature domains, fusion methods can leverage the strengths of each, thereby improving the quality and accuracy of image generation. For example, Li et al. [31] proposed a Laplacian redecomposition framework that effectively addresses issues such as color distortion, blurring, and noise in multimodal medical image fusion by fusing redundant and complementary information. Han et al. [32] proposed an edge-guided adversarial network framework, which optimizes image content and structural information through an edge fidelity constraint, significantly improving remote sensing image compression quality. Additionally, Han et al. [33] introduced a progressive feature interleaved fusion framework that combines convolutional neural networks and transformers to enhance the accuracy of salient object detection in complex backgrounds. Zhang et al. [34] proposed a transformer-based conditional generative adversarial network framework, which uses adversarial training and multi-scale fusion modules to address the issues of long-distance dependencies and prior knowledge integration, significantly improving image fusion results. These studies demonstrate the wide application and effectiveness of fusion methods in image generation.

However, despite the significant progress made by existing fusion methods in various fields, generating tissue slide images still faces several challenges. In tissue slide image generation, the images contain complex details and structures, particularly the subtle differences between different types of tissue and complex boundaries, making it difficult for existing fusion strategies to simultaneously maintain global consistency and local fine structures.

## Proposed method

In this section, we provide a detailed explanation of the four key components of the proposed method: generator architecture, variable window mixing attention module, spectral filtering module, cross-attention fusion module, and loss function. Each part is further elaborated, with emphasis on their specific functions and the critical roles they play in generating high-quality images.

### Generator architecture

**Fig. 2:**
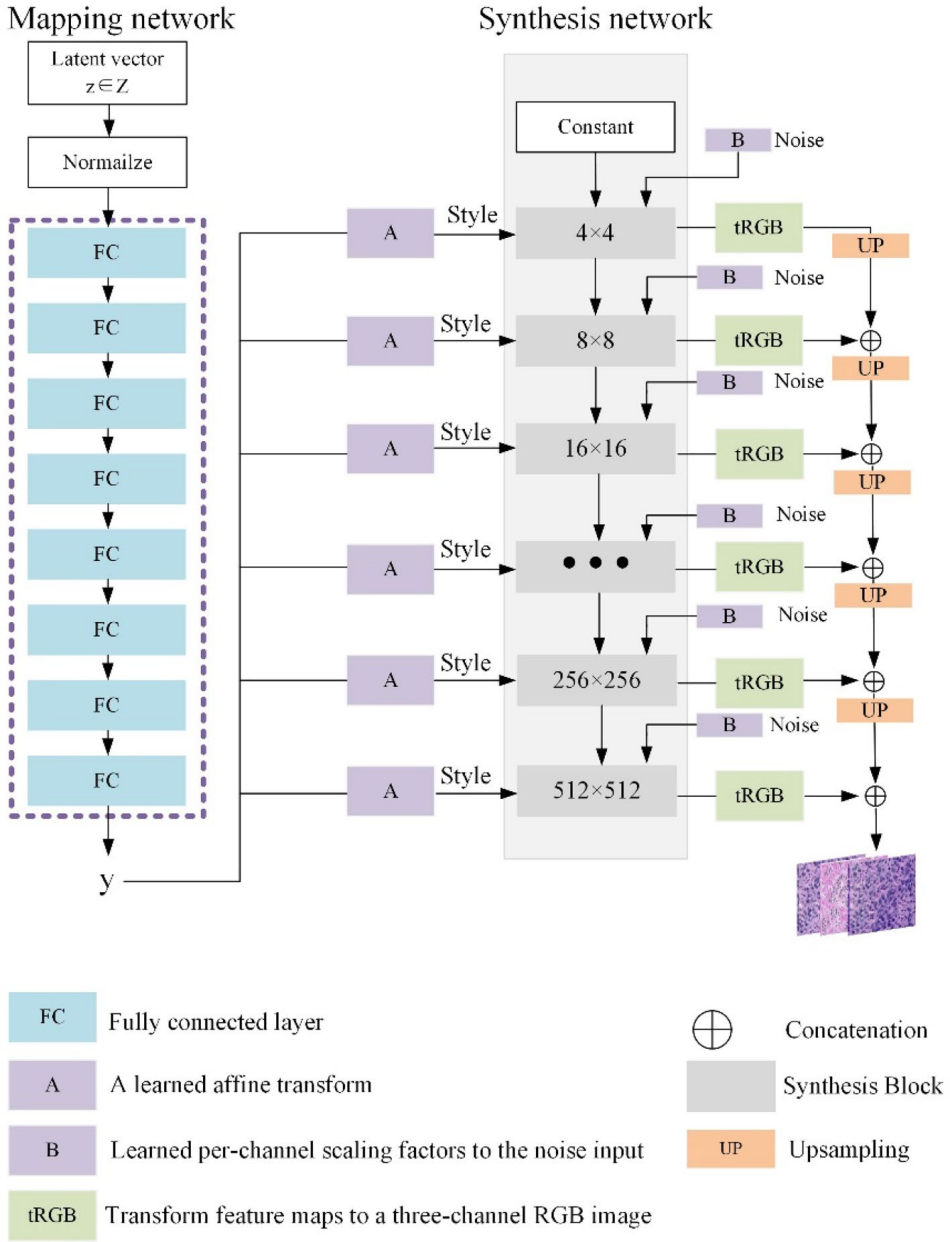
The generator structure consists of two parts, the mapping network and the synthesis network. The mapping network is responsible for generating style vectors and injecting them into various layers of the synthesis network to control the style attributes of the generated image. The synthesis network is tasked with generating tissue slice images layer by layer from low resolution to high resolution

We propose a generator architecture based on StyleGAN [35], as illustrated in Fig. 2. This architecture consists of two main modules, the style generation network and the synthesis network. In the style generation network module, the input latent vector is mapped to an intermediate latent space by the mapping network, and then injected into various layers of the synthesis network through the style modulation network to control the style attributes of the generated image. The synthesis network module generates images layer by layer from low resolution to high resolution. The implementation processes of these two modules are described in detail in the content in this section.

We first input a $512 \times 1$ dimensional latent code into a mapping network composed of eight fully connected layers and obtain an output $y$ of the same dimension as shown in Eq. (1), where $f_{{{\text{map}}}} ( \cdot )$ represents the mapping network. The aim of this operation is to allow the generator to better disentangle features and enrich the diversity and details of the generated images. Through this mapping, the network can learn a more disentangled latent representation, thereby improving the quality of the generated images and allowing for finer control over different features of the image.

1 $$y = f_{{{\text{map}}}} (z).$$

Subsequently, the style vector is injected into feature maps at different resolutions to modulate the style of each layer’s feature map. This operation is implemented using Adaptive Instance Normalization (AdaIN), as described below:

2 $${\text{AdaIN}}\;(X_{i} ,y_{i} ) = y_{s,i} \left( {\frac{{X_{i} - \mu (X_{i} )}}{{\sigma (X_{i} )}}} \right) + y_{b,i} ,$$

In this process, $X_{i}$ represents the feature map of the $i - th$ synthesis block, and $y_{i}$ is the style vector for the $i - th$ layer, which is derived from the mapped latent vector$y$. The terms $\sigma \;( \cdot )$ and $\mu \;( \cdot )$ denote the mean and standard deviation of the feature map, respectively, while $y_{s,i}$ and $y_{b,i}$ represent the scaling factor and bias of the feature map at the $i - th$ block, which are learned separately by two distinct fully connected networks. To ensure that noise only affects subtle variations in the image style, a scaled noise is added to each channel before the ${\text{AdaIN}}$ module. This slight alteration in the visual expression of features at different resolution levels often results in generated images that are more realistic and diverse.

### Variable window mixing attention module

At each resolution stage, we propose a feature extraction strategy using local window attention and varied-size window attention. In generating high-resolution images, different parts of the image often have significant correlations, and using a self-attention mechanism can help capture and maintain critical long-distance dependencies, thus producing more realistic and consistent images. For example, in tissue slides, the positional relationships between nuclei and cytoplasm are closely related, and the transition areas between tumor cells and normal cells also represent important long-distance information. However, feature extraction modules based on self-attention mechanisms tend to have high computational complexity [13], with a complexity of $O\;(n^{2} )$, where $n$ is the spatial dimension of the input feature map. Liu et, al [36] proposed a local window attention mechanism to effectively reduce computational complexity, but it has certain limitations, such as its inability to effectively capture long-distance dependencies in images. Therefore, we propose a hybrid attention module based on both local fixed window attention and variable window attention mechanisms, which reduces computational complexity and effectively captures long-distance dependencies in images.

First, we use $X_{w}$ and $X_{v}$ to represent image blocks divided under fixed and variable window partitions, respectively and $X_{w} ,X_{v} \in R^{{\frac{HW}{{k_{i}^{2} }} \times k_{i} \times k_{i} \times C}}$. In this context, $k_{i}$ represents the size of the window, while $H$, $W$, and $C$ respectively represent the height, width, and number of channels of the feature map. For $X_{w}$, $k_{i}$ is a fixed value; for $X_{v}$, $k_{i}$ is a variable value. Subsequently, we calculate the attention heads for each method using two different attention mechanisms, as shown in Eq. (3).

3 $$head_{i} = \left\{ {\begin{array}{*{20}l} {{\text{LWA}}(X_{w} W_{i}^{Q} ,X_{w} W_{i}^{K} ,X_{w} W_{i}^{V} )} \hfill & {i < \left\lfloor \frac{h}{2} \right\rfloor } \hfill \\ {} \hfill & {} \hfill \\ {{\text{VSA}}(X_{v} W_{i}^{Q} ,X_{v} W_{i}^{K} ,X_{v} W_{i}^{V} )} \hfill & {i > \left\lfloor \frac{h}{2} \right\rfloor } \hfill \\ \end{array} } \right.,$$

Here, $W^{Q}$, $W^{K}$, and $W^{V}$ represent the weight matrices for queries, keys, and values in the self-attention mechanism, each belonging to $R^{C \times (C/h)}$, where $h$ is the number of attention heads. ${\text{LWA}}\;( \cdot )$ and ${\text{VSA}}\;( \cdot )$ stand for local window attention and varied-size window attention, respectively. Subsequently, we propose a hybrid attention mechanism that enables a single transformer module to capture contextual information from both local and varied-sized windows, thus generating the final output $X_{1}$ as shown in Eq. (4):

4 $$X_{1} = {\text{Concat}}\;(head_{1} ,...,head_{h} )\;W^{O} .$$

**Fig. 3.**
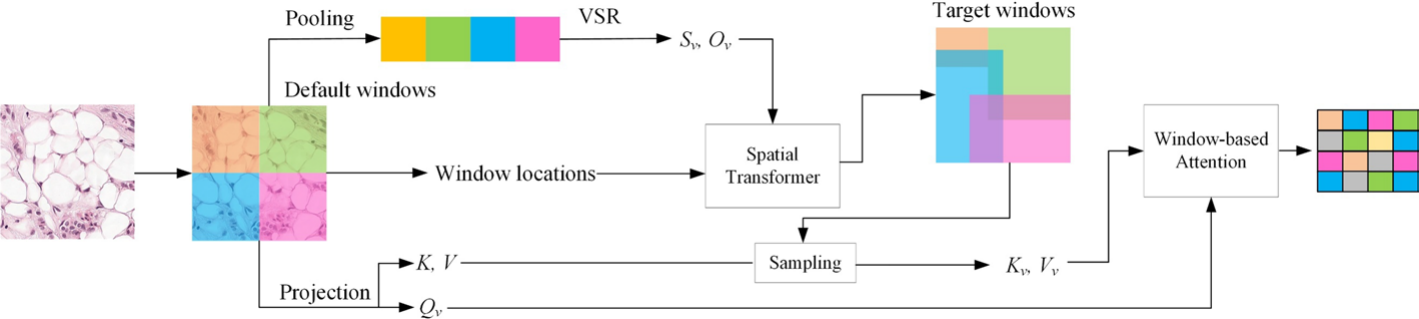
Varied-size window attention mechanism. First, images are divided into default windows, then simplified through pooling and transformed into query vectors ($Q_{v}$), keys ($K$), and values ($V$). The varied-size window regression (VSR) module adjusts the size and position of the windows to create target windows and using the keys and values from the target windows, calculate the attention weights to generate the attention map

Here, $W^{O} \in R^{C \times C}$ represents the projection matrix used to obtain the output from the hybrid attention heads. The computation method for varied-size window self-attention differs from that of fixed window self-attention, with the specific implementation process illustrated in Fig. 3. For a given input, the image is first divided into multiple windows $X_{\nu }$ according to a predefined window size $k$, and the query features $Q_{\nu }$ are obtained based on a linear transformation, as shown in the following equation:

5 $$Q_{\nu } = {\text{Linear}}\;(X_{\nu } ).$$

Subsequently, to estimate the size and location of the target window corresponding to each predefined window, we employ a varied-size window regression module to estimate the size and position of the reference window. This module includes a Leaky ReLU [37] activation layer, a 1 × 1 convolutional layer with a stride of 1, followed in sequence, and an average pooling layer based on the default window size and stride, as illustrated in Eq. (6):

6 $$S_{\nu } ,O_{\nu } = {\text{Conv}} \circ {\text{Leaky}}\;{\text{ReLU}} \circ {\text{Average}}\;{\text{Pool}}(X_{\nu } ),$$

Here, $S_{\nu } ,O_{\nu } \in R^{2 \times h/2}$ represent the estimated scale ratios and offsets in the horizontal and vertical directions, respectively. Subsequently, we obtain the global key and value from the input feature map $X$, as shown in Eq. (7):

7 $$K,V = {\text{Reshape}} \circ {\text{Linear}}\;(X),$$

**Fig. 4.**
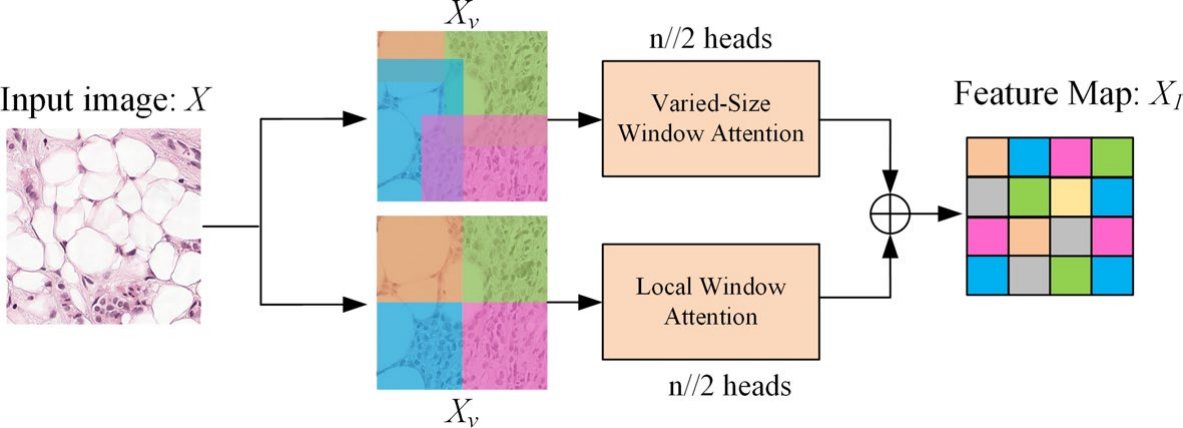
Structure of the variable window mixing attention module. The input image *X* is divided into two parts, each processed by half of the attention heads. The left part, *X*_*v*_, uses a varied-size window attention mechanism to dynamically segment the image, while the right part, *X*_*w*_, uses a local window attention mechanism to extract local features. Both modules calculate mixed attention based on object size and local details, and the combined outputs produce the final image

Here, the key and value $K$ and $V$ belong to $R^{H \times W \times C}$. Finally, the varied-size window attention module uniformly samples $M$ features from each window of different sizes on $K$ and $V$, obtaining the keys and values based on different windows, denoted as $K_{V}$ and $Q_{V}$ respectively, and together they are used to calculate the attention weights. The structure of Variable Window Mixing Attention Module is shown in Fig. 4

### Spectral filtering module

**Fig. 5.**
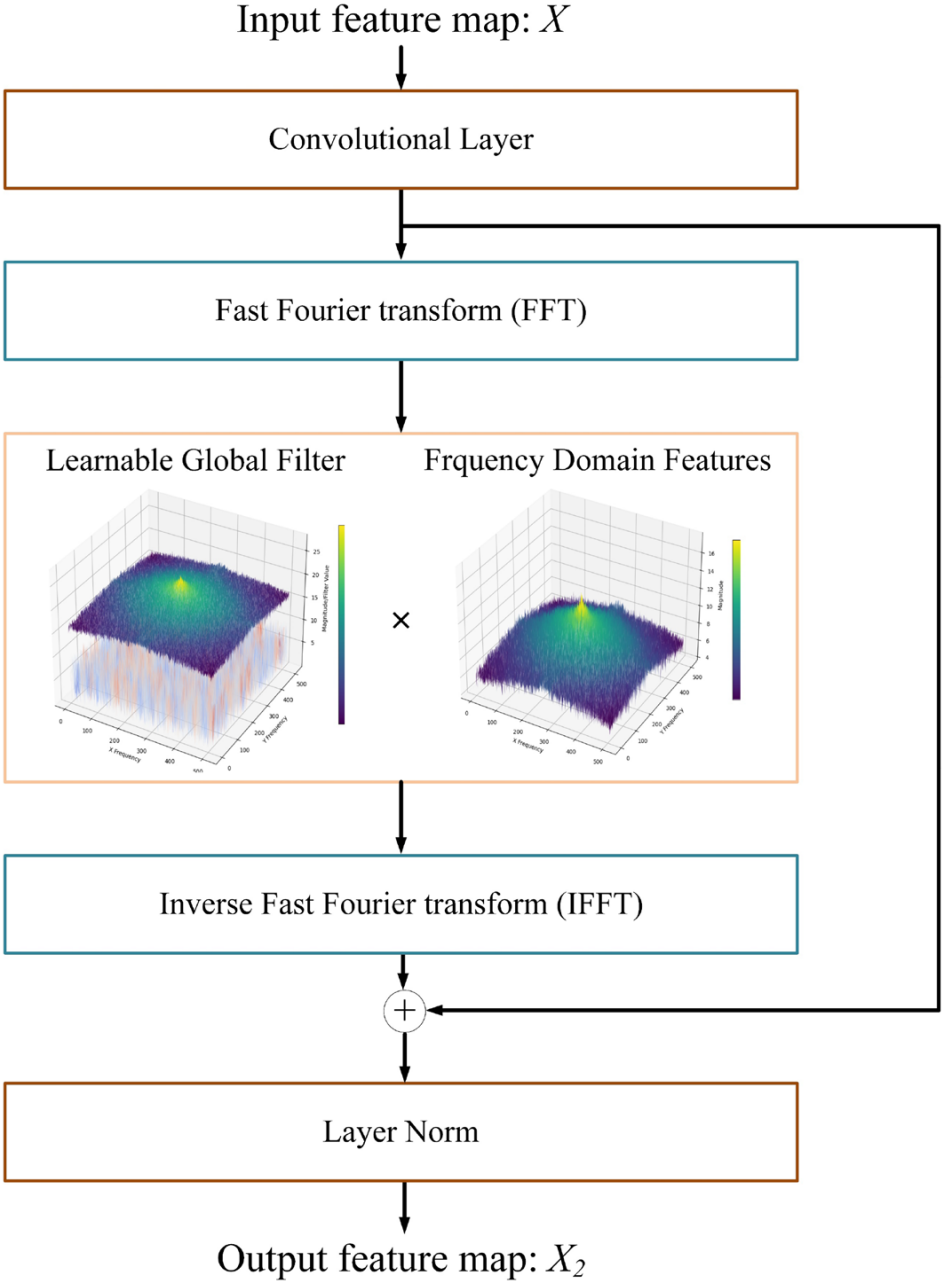
Structure of the spectral filtering module. The input feature map X, after feature extraction via a convolutional layer, is transformed to the frequency domain through Fast Fourier Transform (FFT), where it is element-wise multiplied with a learnable global filter. Then, the data is converted back to the spatial domain using Inverse Fast Fourier Transform (IFFT). Finally, through layer normalization, the training process is stabilized, producing the final output

In the process of generating histological images, certain structural patterns, such as cell textures, symmetrical structures, and multi-scale patterns, exhibit periodic or repetitive features. The spectral filtering module effectively captures these periodic features, enhancing the model's ability to understand the global structure of the image, the structure of the module is shown in Fig. 5. First, spatial domain features are transferred into the frequency domain through a Fast Fourier Transform [38]. Subsequently, a learnable filter is used to select frequency domain features, automatically identifying and emphasizing the important information by assigning higher weights to these important features. Meanwhile, for less important or redundant frequency domain information, the filter assigns lower weights or suppresses it appropriately. Finally, the processed frequency domain data is converted back to the spatial domain through an inverse Fast Fourier Transform, ensuring that the final output maintains the integrity and practicality of the image content. The specific equation is as shown in Eq. (8):

8 $$X^{\prime} = {\mathcal{F}}[X] \in C^{H \times W \times C} ,$$

Here, ${\mathcal{F}}\,[ \cdot ]$ represents the Fast Fourier Transform, and $X$ denotes the feature map input for this layer, with dimension $C^{H \times W \times C}$. Subsequently, a global filtering operation is applied to $X$, resulting in the modulated spectrum $\tilde{X}$, as shown in Eq. (9):

9 $$\tilde{X} = G \odot X^{\prime},$$

where $\odot$ is the element-wise multiplication, $G$ is a learnable global filter. Finally, $\tilde{X}$ is subjected to an inverse Fast Fourier Transform to revert it back to the spatial domain, as shown in Eq. (10):

10 $$X_{2} \leftarrow {\mathcal{F}}^{ - 1} [\tilde{X}],$$

Here, ${\mathcal{F}}^{ - 1} [ \cdot ]$ represents the inverse Fast Fourier Transform, and $X_{2}$ denotes the output of spectral filtering module.

### Cross-attention fusion module

**Fig. 6:**
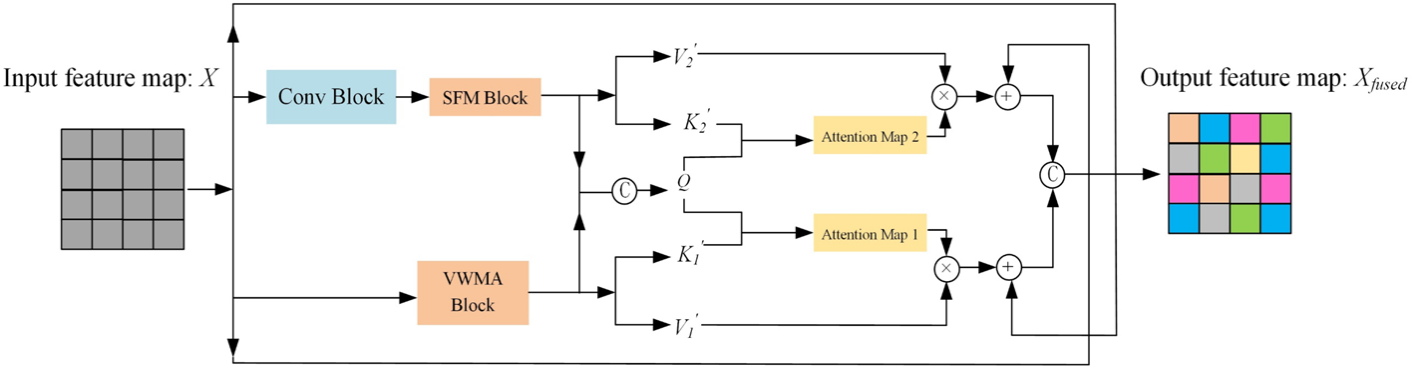
This diagram depicts the synthesis block with cross-attention fusion module. Input data is processed through a Variable Window Mixing Attention Block (VWMA) and a Convolutional Block (Conv), generating features that are refined by a Spectral Filtering Module (SFM) to create attention maps. These maps combine with values to form weighted features, which are merged and enhanced in a fusion layer to produce the final output, optimizing the model's attention mechanism efficiency

When handling complex spatial structures, using variable window mixing attention module alone may not achieve the direct efficiency of Convolutional Neural Networks, and relying solely on the spectral filtering module might not effectively capture long-distance dependencies. Therefore, we propose the cross-attention fusion module that combines the advantages of attention mechanisms and frequency domain filtering. Unlike simple concatenation fusion methods, which often lead to information redundancy by directly connecting features from both domains and potentially retaining repetitive or irrelevant information, our proposed fusion method first performs an initial fusion of the dual-domain features, combining spatial and frequency domain features to ensure the preliminary integration of important information. Next, we apply a cross-attention mechanism to further compute and optimize the information from both domains and the single domain. Specifically, the cross-attention mechanism allows each domain to be guided by the information from the other domain, thereby enhancing feature extraction within the single domain and integrating key information from the other domain. This enables the model to focus more precisely on the important features in the single domain, avoid introducing redundant information, and improve the effectiveness and accuracy of feature extraction. Finally, the information from the two single domains is fused to generate higher-quality images. This mechanism not only avoids the noise and redundant information that may be introduced by traditional concatenation methods but also fully leverages the complementary strengths of spatial and frequency domain features, leading to more precise feature fusion and significantly improving the overall model performance. As shown in Fig. 6, each layer’s input first passes through the variable window mixing attention to obtain an intermediate output $X_{1}$, and then through the spectral filtering module to obtain an intermediate output $X_{2}$. Subsequently, we use cross-attention mechanisms and residual connections to guide and learn from these two types of features, thereby obtaining a comprehensive output that includes multi-domain information. Such a design not only enhances the depth and breadth of feature processing but also optimizes the overall performance of the model. The specific equations are as follows:

11 $$K_{j}^{\prime } = {\text{Reshape}}\;({\text{Conv}}_{K}^{j} (X_{j} )),$$

12 $$V_{j}^{\prime } = {\text{Reshape}}\;({\text{Conv}}_{V}^{j} (X_{j} )),$$

Here, $j = 1,2$, $K{\prime}_{j} \in R^{H \times W \times C}$ represent the key, $V{\prime}_{j} \in R^{H \times W \times C}$ represents the value,.${\text{Conv}}\;{(} \cdot {)}$ represents a convolutional layer with a 3 × 3 kernel size, and ${\text{Reshape}}\;{(} \cdot {)}$ represents the reshape operation. Subsequently, by computing the query and obtaining the attention scores, combined with residual connections, the final output $X_{fused}$ is produced, as shown in the following equations:

13 $$Q{\prime} = {\text{Reshape}}({\text{Conv}}({\text{Concat}}(X_{1} ,X_{2} )),$$

14 $$A_{j}^{\prime } = {\text{Softmax}}(Q_{j} K_{j}^{T} ),$$

15 $$X_{fused} = {\text{Conv}}\;({\text{Concat}}\;(X_{2} \; \oplus \;{\text{Reshape}}\;(A_{1}^{\prime } V_{1}^{\prime } ),\,\;X_{1} \; \oplus \;{\text{Reshape}}\;(A_{2}^{\prime } V_{2}^{\prime } ))).$$

Here, $Q{\prime} \in R^{H \times W \times C}$ represents the domain-invariant query, allowing us to leverage attributes from different domains for complementation. It is worth noting that both frequency domain and spatial domain features are integrated to produce the domain-invariant query, enabling us to fully utilize the complementary attributes across different domains. $A^{\prime}_{j} \in R^{HW \times HW}$ represents the attention map of different domains; $\oplus$ indicates element-wise addition of matrices, $X_{fused}$ denotes the output corresponding to the resolution of that layer.

### Loss function

The network is trained using non-saturating logistic GAN loss and the R1 gradient penalty term is applied to suppress mode collapse and promote the generator to learn more realistic image features. The loss functions are specifically expressed as follows:

16 $${\mathcal{L}}_{D} = - {\mathbb{E}}_{{x\sim P_{x} }} \left[ {log\;(D(x))} \right] - {\mathbb{E}}_{{x\sim P_{x} }} \left[ {log(1 - D\;(G(z)))} \right] + \gamma \cdot {\mathbb{E}}_{{x\sim P_{x} }} \left[ {\left\| {\nabla_{x} D\;(x)} \right\|_{2}^{2} } \right],$$

17 $${\mathcal{L}}_{G} = - {\mathbb{E}}_{{z\sim P_{z} }} [log\;(D\;(\;G(z)))].$$

For the discriminator loss ${\mathcal{L}}_{D}$, ${\mathbb{E}}_{{x\sim P_{x} }} [log\;(D\;(x))]$ represents the expected output of the discriminator for real data. The discriminator aims for $D\;(x)$ to be as close to 1 as possible, ${\mathbb{E}}_{{x\sim P_{x} }} [log\;(1 - D\;(G(z)))]$ represents the output of the discriminator for generated data, the discriminator aims for $D\;(G\;(z))$ to be as close to 0 as possible, $\gamma \cdot {\mathbb{E}}_{{x\sim P_{x} }} [\left\| {\nabla_{x} D(x)} \right\|_{2}^{2} ]$ is used for gradient penalty on the discriminator to prevent overfitting and increase the diversity of generated tissue.

slide images, where $\gamma$ is a hyperparameter, $\nabla_{x} D(x)$ represents the squared norm of the discriminator's gradient on real data $x$. For the generator loss ${\mathcal{L}}_{G}$, ${\mathbb{E}}_{{z\sim P_{z} }} [log(D(G(z)))]$ represents the expected output of the generator for generated data. The generator aims for $D(G(z))$ to be as close to 1 as possible.

### Network structure

The intricate configuration of the generator is delineated in Table 1. Ranging from 4 × 4 to 32 × 32 resolution, the model generates images through the utilization of a cross-attention fusion module, encompassing 512 channels, window sizes of 4 × 4 and 8 × 8, and 16 attention heads, while preserving the output channels at 512. Furthermore, upsampling procedures are executed at every resolution phase, ensuring consistency in channel quantity. Upon reaching the 64 × 64 resolution phase, the channel quantity of the cross-attention fusion module is halved to 256, featuring a window size of 8 × 8, and a reduction in the output channel count to 256. After upsampling, the channels are further diminished to 128. Progressing from 128 × 128 to 512 × 512 stages, the model exclusively employs the variable window mixing attention module, witnessing a decline in channel counts from 128 to 32 as dimensions amplify, while maintaining the window size and attention heads at 8 × 8 and 4, respectively. The channels undergo additional reductions with each successive step of upsampling. We also conducted an efficiency analysis of the various components of the model, as shown in Table 2.

**Table 1:** The detailed structure of generator

Input size	Layer configuration details
4 × 4	{Cross attn, in = 512, window = 4 × 4, 16 heads, out = 512} × 2
	Upsampling, channels = 512
8 × 8	{Cross attn, in = 512, window = 8 × 8, 16 heads, out = 512} × 2
	Upsampling, channels = 512
16 × 16	{Cross attn, in = 512, window = 8 × 8, 16 heads, out = 512} × 2
	Upsampling, channels = 512
32 × 32	{Cross attn, in = 512, window = 8 × 8, 16 heads, out = 512} × 2
	Upsampling, channels = 256
64 × 64	{Cross attn, in = 256, window = 8 × 8, 8 heads, out = 256} × 2
	Upsampling, channels = 128
128 × 128	{VAM attn, in = 128, window = 8 × 8, 4 heads, out = 128} × 2
	Upsampling, channels = 64
256 × 256	{VAM attn, in = 64, window = 8 × 8, 4 heads, out = 64} × 2
	Upsampling, channels = 32
512 × 512	{VAM attn, in = 32, window = 8 × 8, 4 heads, out = 32} × 2

**Table 2:** The model efficiency analysis

	Complexity (FLOPs)	#Parameters
Self-Attention	*O* (HWD^2^ + H^2^W^2^D)	4D^2^
Window-Attention	*O* (HWD^2^ + M^2^HWD)	4D^2^
Spectral Filter	*O* (HWD [log_2_(HW)] + HWD)	HWD
Cross-Attention Fusion	*O* (HWD^2^ + H^2^W^2^D)	8D^2^

H represents the height, W represents the width, and D represents the number of channels of the feature maps, M represents the size of local window

## Experiments

### Experiment settings

In this section, we provide a detailed explanation of our experimental setup. Specifically, we comprehensively validated our method on the PCAM200 dataset using three image generation quality evaluation metrics and two no-reference image quality assessment metrics. Additionally, we compared the proposed method with eight other tissues slide image generation methods. Below, we provide a detailed introduction to the dataset used in this study, the comparison methods selected, and the evaluation metrics used to assess the quality of the generated images.

### Dataset

The PCAM 200 dataset [39] is a public histological image dataset provided by Patch Camelyon, created in the same manner as the Camelyon2016 challenge [40] dataset. This dataset contains 327,680 color images with a resolution of 512 × 512 pixels. Each image is labeled with a binary label, indicating either tumor or normal. In our experiments, we used a specific subset of the dataset. Specifically, we randomly selected 2000 tissue slide images from the overall training data, including 1000 labeled as “tumor” and 1000 labeled as “normal”. This selection method is designed to ensure a balanced dataset in terms of categories, allowing the model to learn and distinguish between the two different types of tissue features more accurately. In addition, the random selection method helps to increase the representativeness and diversity of the data, making the training process more efficient and potentially enhancing the generalization ability of the model.

### Compared methods

In comparative experiments, we used the PCAM200 dataset as a benchmark to comprehensively evaluate eight predominant unsupervised image generation methods. These methods include GAN [41], GANformer [42], StyleGAN2 [43], ProjectedGAN [44], LightweightGAN[45], Wavediff [46], LFM [47] and RDUOT [48]. The purpose of this comparison is to conduct an in-depth analysis and evaluation of the effectiveness and applicability of these advanced methods, as well as the proposed method, in generating tissue slide images.

### Evaluation metrics

In evaluating the technology for generating histopathological tissue slide images, we used five image generation quality evaluation metrics for a comprehensive quantitative assessment of the generated image quality. Fréchet Inception Distance (FID) [49] measures the difference between the generated images and real images in the feature space by calculating the Wasserstein distance between the features extracted by a pre-trained Inception v3 network. A lower FID value indicates greater similarity between the generated images and real images. Inception Score (IS) [50] which assesses the quality and diversity of generated images by classifying them using the Inception v3 network and calculating the confidence of the classifications and the entropy of the image class distribution. A higher IS value indicates higher image quality and diversity. Kernel Inception Distance (KID) [51] which uses the Inception v3 pre-trained model and multiple kernel maximum mean discrepancy to measure the difference between generated images and real images. A lower KID value indicates greater similarity between the generated images and real images. MA Score [52] provides a comprehensive assessment of the generated images, with higher scores indicating higher visual quality and closer alignment with human perception standards. Natural Image Quality Evaluator (NIQE) [53] is a no-reference image quality assessment metric based on a statistical model, evaluating quality by measuring the naturalness and statistical properties of the images. A lower NIQE value indicates better image quality. These evaluation metrics comprehensively consider the similarity in distribution between generated and real images, as well as characteristics such as clarity, contrast, and naturalness of the generated images, providing a thorough reference for assessing the quality of tissue slide images.

### Qualitative and quantitative comparisons

#### Qualitative Comparisons

**Fig. 7:**
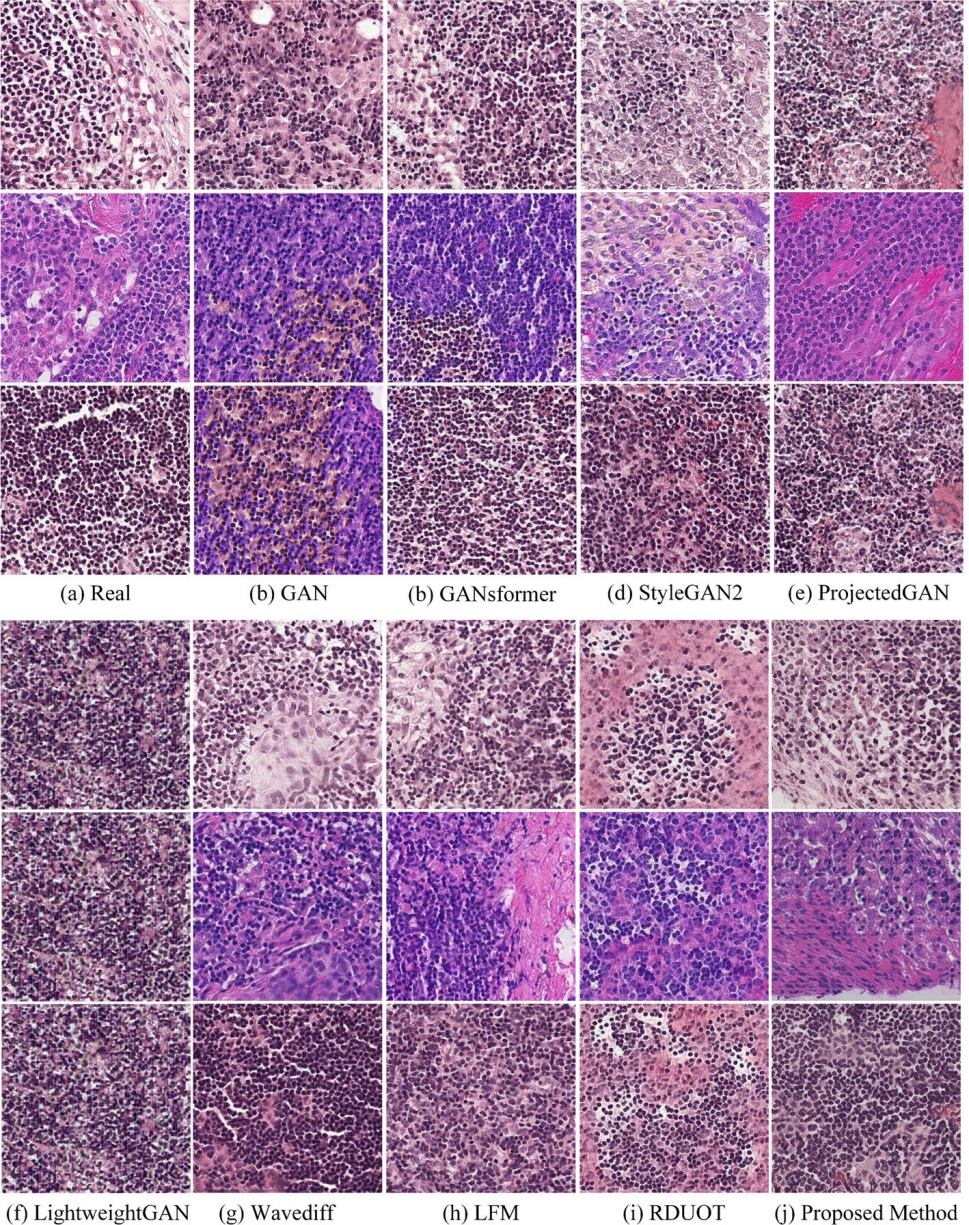
Comparison of histological image generation results between the proposed method and eight other deep learning-based techniques. **a** Real image. **b** GAN [42]. **c** GANsformer [43]. **d** StyleGAN2 [44]. **e** ProjectedGAN [45]. **f** LightweightGAN [46]. **g** Wavediff [47]. **h** LFM [48]. (i) RDUOT [49] (j) Proposed Method

Figure 7 shows the qualitative comparison between the proposed method and eight other methods. (a) represents real tissue slide images, (b) represents images generated by GAN. In the tissue slides generated by this method, the cell nuclei are overly stained, leading to unclear details such as the nuclear membrane and nucleoli. Additionally, there is an excess of hematoxylin in cytoplasm, causing an imbalance between the nuclei and cytoplasm. (c) represents tissue slides generated by GANformer. These generated slides suffer from insufficient transparency, resulting in slightly blurred image details. Moreover, the cytoplasm color is relatively light, lacking the color saturation found in real slide images, making the overall visual effect less vivid and striking. (d) represents tissue slide images generated by StyleGAN2. It can be observed that in these generated images, the cell nuclei are too lightly stained, while the cytoplasm is overly stained, leading to insufficient differentiation. The overall contrast between the nuclei and cytoplasm is poor, making the boundary between the nuclei and cytoplasm unclear, which affects the visual effect and diagnostic accuracy of the images. (e) represents tissue slide images generated by ProjectedGAN. In these images, the cell structures are not clear enough, some tissues are incomplete, the staining is uneven, and there are impurities after staining. (f) represents tissue slide images generated by LightweightGAN. These images exhibit obvious mode collapse, with the images generated lacking diversity, leading to high repetition in cell structures and morphology, failing to adequately reflect the complexity and diversity of real tissue slides. (g) represents tissue slide images generated by Wavediff, some areas exhibit blurring, and the nuclear structures are not clearly defined. (h) represents tissue slide images generated by LFM, there is uneven staining, and some tissues appear blurred. Overall, these methods have certain issues and clear deficiencies compared to our proposed method (i). The tissue slides generated by RDUOT exhibit a mist-like artifact, which significantly impairs the clarity of the cellular structure, making it extremely difficult to observe and analyze the details. (j). The proposed method outperforms in staining quality, with generated slides showing complete tissues under the microscope, good transparency, and cytoplasmic contrast, and clear backgrounds without contamination or bubbles. Specifically, in these generated images, the red contrast between the nuclei and cytoplasm is clear and distinct, the cell contours and structures are complete, the colors are bright, and the contrast is excellent. These characteristics ensure the clarity of the slide images under the microscope, aiding accurate judgment by the viewers.

#### Quantitative comparisons

When comprehensively evaluating the quality of generated histological slide images, we conducted a systematic analysis using a series of quantitative metrics. Specifically, we compared eight different tissues slide generation methods on the PCAM 200 dataset using three image generation evaluation metrics and two no-reference image quality assessment metrics. These evaluation metrics include FID, IS, KID for generated image quality, as well as MA and NIQE for no-reference image quality. Through comprehensive analysis of these metrics, we can thoroughly evaluate the strengths and weaknesses of each generation method in terms of image quality, ensuring the objectivity and accuracy of the comparison results.

As shown in Table 3, in the comparative experiments, our proposed method performed the best in terms of the FID metric, achieving the lowest FID value. This indicates that our proposed method outperforms in the similarity between generated images and real images, with the smallest difference in feature space, thus producing the highest quality images. Regarding the Inception Score metric, our method achieved an IS value of 2.0171, second only to ProjectedGAN’s 2.4620. This suggests that our generated images perform well in terms of quality and diversity, with high classification confidence and even class distribution. For the KID metric, our method achieved a value of 0.0151, the best among all comparison algorithms, demonstrating the highest similarity between our generated images and real images in feature space. This shows that the proposed method performs very well in capturing the details and feature distribution of the image. In the two no-reference image quality assessment indicators, the proposed method achieves a MA value of 7.2831, indicating that the generated image has the best subjective quality. The NIQE value of the proposed method is 4.9504, which is second only to StyleGAN2's 4.6916, indicating that the naturalness and statistical properties of the generated images are very close to real images. These comprehensive evaluation results show that the proposed method not only performs well in a single metric, but also shows overall superiority in multiple evaluation dimensions, which verifies the effectiveness and superiority of the proposed method in practical applications. These results fully demonstrate the great potential of the proposed method in generating high-quality histological tissue slide images, which provides a solid foundation for future medical image generation and analysis.

**Table 3:** Comparison of generated images results between the proposed method and eight other methods, GAN [41], GANformer [42], StyleGAN2 [43], ProjectedGAN [44], LightweightGAN [45], Wavediff [46], LFM [47] and RDUOT [48]

Method/Metrics	FID↓	IS↑	KID↓	MA↑	NIQE↓
GAN[41]	60.2101	1.8890	0.0298	7.1125	6.8611
GANsFormer[42]	55.3206	1.8058	0.0273	7.0167	5.9035
StyleGAN2[43]	47.4542	1.8383	0.0231	7.0102	**4.6916**
ProjectedGAN[44]	49.0065	**2.4620**	0.0210	6.5397	6.6792
LightweightGAN[45]	285.977	0.2983	1.0207	5.2040	7.8127
Wavediff [46]	48.7622	1.7350	0.0204	6.6743	6.0625
LFM [47]	59.8524	1.8569	0.0409	6.6510	6.0993
RDUOT [48]	114.920	1.6660	0.0885	6.4736	5.3790
Proposed method	**41.8336**	2.0171	**0.0151**	**7.2831**	4.9504

The best result is bold, and the second-best result is underlined

### Ablation study

In this section, we deeply analyzed the effectiveness of the proposed variable window mixing attention module and spectral filtering module on the PCAM200 dataset through qualitative and quantitative ablation experiments. By removing the variable window mixing attention module, its impact on the overall performance of the method is evaluated, which effectively proves the important role of the variable window mixing attention module in enhancing the quality of the generated tissue slide images. Similarly, by removing the spectral filtering module, the importance of the spectral filtering module in making full use of the image frequency domain information to improve the image quality is further analyzed. Finally, the variable window mixing attention module, and the spectral filtering module were removed to evaluate the overall impact of their simultaneous absence on the quality of the generated images and the performance of the algorithm. These experimental results clearly demonstrate the critical role of each module in our method, proving their contribution to improving the quality of the generated images and the overall performance of the algorithm.

**Fig. 8:**
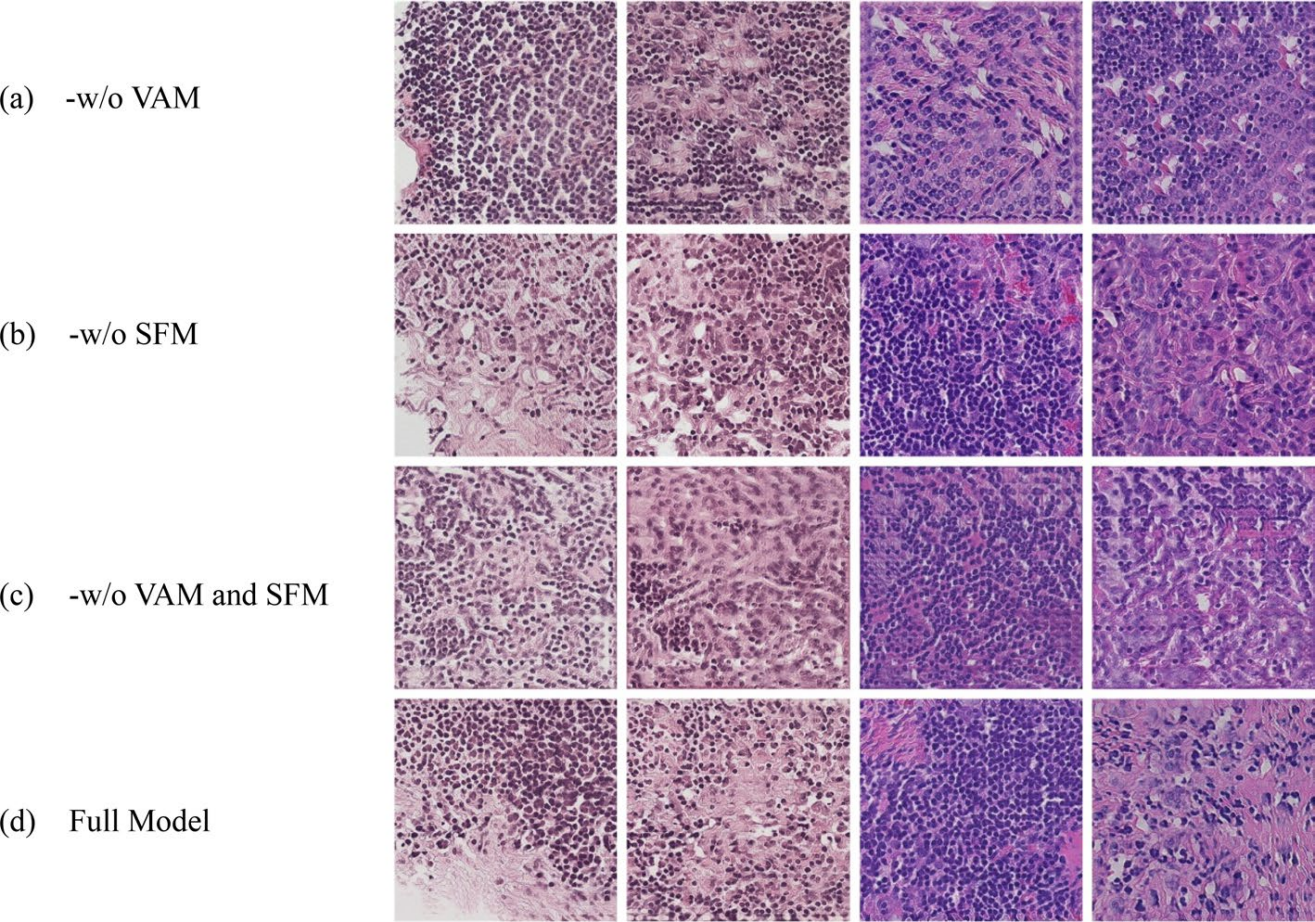
Qualitative ablation experiment visualization **a**-w/o VAM denotes the images generated by the method without the variable window mixing attention module; **b** -w/o SFM represents the images generated by the method without the spectral filtering module; **c** -w/o VAM and SFM denotes the images generated by the method without VAM and SFM; **d** Full Model represents the images generated by the full method

Firstly, we conducted qualitative comparison experiments. In Fig. 8, (a) “- w/o VAM” represents the method without the variable window mixing attention module, showing the generated image effects the spectral filtering module. (c) “- w/o VAM and SFM” represents the tissue slides generated by the method with both the VAM and the SFM removed. (d) “Full model” represents the images generated by the method containing all modules. The comparative analysis reveals that removing any key module leads to a significant decline in model performance. Specifically, the tissue slides images generated without the variable window mixing attention module exhibit the nuclear cytoplasm shows clumped distribution, the cytoplasmic color is uneven, and some parts are overly light and overlap with the background, indicating that the VAM module plays a crucial role in image feature extraction and detail preservation. The tissue slides images generated without the spectral filtering module show the cytoplasm that appears mottled red, and the cells under the microscope are blurry with low resolution, suggesting that the SFM module is critical for capturing image frequency domain information and enhancing the naturalness of the images. The images generated with both the variable window mixing attention module and the spectral filtering module removed exhibit some of the nuclei are reddish, blending homogeneously with the eosinophilic cytoplasm. In contrast, the images generated by the full model are noticeably superior to those generated by any ablated model, demonstrating the contrast between the nucleus and cytoplasm is distinct, with clear quantity and structure.

Subsequently, we conducted quantitative comparison experiments. Table 4 presents the results of the ablation experiments of the proposed method on the PCAM 200 dataset. By removing key modules and using the five image quality evaluation metrics mentioned earlier, we can precisely reveal the contribution and effectiveness of each module to the overall algorithm. As shown in Table 4, the full model achieves the best performance across all metrics, indicating that the proposed method has a significant advantage in overall performance. The collaboration of all modules in the model enhances the overall quality of the generated images and the robustness of the algorithm.

**Table 4:** The results of the ablation experiments of the different modules

Ablated Models	FID↓	IS↑	KID↓	MA↑	NIQE↓
-w/o VAM	42.9043	2.0849	0.0147	7.2745	4.8824
-w/o SFM	61.9088	1.9786	0.0298	6.9270	5.5169
-w/o VAM and SFM	58.5471	1.7718	0.0260	7.2555	4.9123
Full model	41.8336	2.0171	0.0151	7.2831	4.9504

## Conclusion

Histological slides are crucial tools in medical diagnostics. By observing tissue slides under a microscope, doctors can diagnose various diseases such as cancer, infections, and inflammation. Generating high-quality histopathological slides requires significant time and human resources and is susceptible to errors and variations during preparation. Utilizing generative technology to create high-quality histopathological slide images can improve the efficiency of medical resource utilization and address issues of insufficient sample quantities and excessive sample variation. To generate high-quality and diverse histological slide images, we propose a generative adversarial network that uses spatial domain and frequency domain information for feature extraction. This method adopts a varied-size window attention mechanism to adaptively adjust the attention window size according to the image content, to better capture local and global information. Fast Fourier transform transforms the image from the spatial domain to the frequency domain, so that the model can process and analyze the frequency component of the image more efficiently. This process can capture high and low frequency information that may be ignored by traditional spatial domain methods, thus enhancing the clarity and detail preservation of the generated images.

Since a single method cannot fully meet the requirements of generating high-quality images, a cross-attention fusion module is introduced in this paper. Our method is not only a simple combination of different techniques, but also an in-depth fusion mechanism to effectively integrate the information of spatial domain and frequency domain, to realize their mutual reference and complementarity, to improve the overall quality and detail performance of the image. Through the cross-attention mechanism, the generated images maintain consistency in the macro structure and show higher fidelity and resolution in the microscopic details. Finally, the proposed method can produce histopathological slide images with high authenticity and diversity, significantly improving the quality and efficiency of medical image analysis. In future work, we plan to further reduce the complexity and parameter count of the model and conduct in-depth optimization of the model structure to meet the requirements for lightweight implementation and intend to extend this study to other image domains, such as medical imaging, remote sensing images, and natural images. Through these efforts, we hope to provide more efficient and accurate image processing and analysis tools for these fields, supporting the progress of scientific research and practical applications.

## Data Availability

The dataset used in this study is the PatchCamelyon (PCAM) dataset, a publicly available dataset for histopathology image classification. The PCAM dataset can be accessed at https://github.com/basveeling/pcam.
